# *TOPBP1 *missense variant Arg309Cys and breast cancer in a German hospital-based case-control study

**DOI:** 10.1186/1477-5751-9-9

**Published:** 2010-11-25

**Authors:** Magda A Blaut, Natalia V Bogdanova, Michael Bremer, Johann H Karstens, Peter Hillemanns, Thilo Dörk

**Affiliations:** 1 Clinics of Obstetrics and Gynaecology, Hannover Medical School, Carl-Neuberg-Str. 1, D-30625 Hannover, Germany; 2 Clinics of Radiation Oncology, Hannover Medical School, Carl-Neuberg-Str. 1, D-30625 Hannover, Germany

## Abstract

The DNA double strand break repair gene *TOPBP1 *has been suggested as a breast cancer susceptibility gene and a missense variant Arg309Cys was observed at elevated frequency in familial breast cancer cases compared to healthy controls from Finland. We found the Arg309Cys allele at a 13% carrier frequency in a hospital-based series of 1064 German breast cancer patients and at a 14% carrier frequency in 1014 population controls (OR 0.89, 95%CI 0.69-1.15; p = 0.4). Arg309Cys carriers were not enriched among patients with a family history of breast cancer (OR = 0.87, 95%CI 0.53-1.43, p = 0.6) and were slightly underrepresented in patients with bilateral disease (OR = 0.49, 95%CI = 0.24-0.99; p = 0.047). In the latter group, the mean age at diagnosis was 62 years in carriers and 54 years in non-carriers (p = 0.004). We conclude that there is no evidence for the TOPBP1*Arg309Cys variant to confer an increased risk for breast cancer in the German population.

## Findings

Only a small proportion of breast cancer cases can be attributed to mutations in high-penetrance genes such as *BRCA1 *or *BRCA2*, and much of the remaining cases are attributed to more common gene variants with lower penetrance [1,2]. As many of the hitherto known susceptibility factors have been implicated in the cellular responses to DNA double-strand breaks and replication stress, there is considerable interest in genetic variants of additional proteins involved in these pathways [2].

*TOPBP1 *encodes a protein that shares homology to BRCA1, is aberrantly expressed in breast carcinomas and has a critical role in DNA damage and replication checkpoint pathways [3-7]. *TOPBP1 *encodes a 1522 amino acid BRCT domain protein that interacts with DNA topoisomerase IIβ and is involved in ATM/ATR-mediated DNA damage and replication checkpoint pathways [3-6]. Reduced or aberrantly localized TopBP1 expression has been observed in a significant proportion of breast cancer specimens [7]. Functional *TOPBP1 *variants therefore represent plausible candidate breast cancer susceptibility alleles. A recent sequencing and case-control association study has assessed the role of germ-line variants in Finnish breast and ovarian cancer patients [8]. The novel Arg309Cys substitution was observed at significantly higher frequency in 125 familial breast and/or ovarian cancer patients compared to 697 healthy controls (15.2% versus 7.0%; P = 0.002), and a 2.4-fold increase in risk was suggested [8]. We aimed to corroborate this finding in a hospital-based series of 1064 German breast cancer patients and 1014 population controls.

Our German study population consisted of a hospital-based series of 1012 unselected breast cancer patients who were treated at the Department of Radiation Oncology at Hannover Medical School from 1996-1999 and an additional small series of 52 patients with bilateral breast cancer collected later from the same hospital. Median age at onset of breast cancer was 57 years in this patient group, and 144 patients (13.5%) reported at least one first-degree relative with breast cancer. The series had been used previously to determine the frequency of mutations in the *BRCA1*, *ATM*, *NBS1 *and *CHEK2 *genes as well as to characterize more common polymorphisms studied by the Breast Cancer Association Consortium. The population controls were 1014 anonymous female German volunteers who had been ascertained between August and December 2005 at Hannover Medical School, Lower Saxony, Germany. Written informed consent had been obtained from all patients, and the study was approved by the local Ethics Commission.

Genomic DNA was isolated from peripheral white blood cells using a routine protocol employing proteinase K digestion and phenol-chloroform extraction. A region spanning the exon 8 was amplified by PCR with the primers 5'-AGATTTCAGTAAACACCCCTG-3' (forward) and 5'-GGTCTTCAAAGTCAGGCTAG-3' (reverse) using HotStart Taq DNA Polymerase (Qiagen) and 35 cycles of 1 min denaturation at 94°C, 1 min annealing at 60°C and 1 min extension at 72°C. 168 samples were initially evaluated by restriction-enzyme based analysis to identify heterozygous and homozygous carriers. For this purpose, PCR products were incubated with *Tat *I (Fermentas) and analysed on a 2% agarose gel. We further established a 5'-nuclease assay for the discrimination of the Arg and Cys alleles using specific FAM- or VIC-labelled MGB probes (Applied Biosystems). The probe sequences were 5'-VIC- TGAAAGAGTACGACCTACA- MGB- 3' and 5'-FAM- TGAAAGAGTACAACCTACA- MGB- 3', respectively. 60 PCR cycles with an annealing step at 60°C were performed in 96-well-plates on a FAST 7500 Sequence Detection System (Applied Biosystems), and genotypes were determined from the fluorescence emission in each well using the FAST 7500 Sequence Detection System software. The call rate was 98.8% in the breast cancer samples and 99.9% in the control series.

To further validate the results of mutation screening, all homozygotes and selected heterozygous samples were confirmed by direct sequencing using a BigDye Terminator protocol and capillary gel electrophoresis on an Avant 3100 Sequence Analyser (Applied Biosystems). Results from direct sequencing, restriction enzyme analysis and from the 5'nuclease assay were concordant in all samples.

Genotype distributions were compared between cases and controls, or between different patient subgroups, and odds ratios were determined under dominant, co-dominant and recessive models using the SNP & Variation Suite 7.0 software (Golden Helix Inc.). Results were considered non-significant for p-values > 0.05 (2 df). Ages at diagnosis were compared using a T-test (Minitab 15) and a median test (Statistix 7.0).

**Figure 1:**
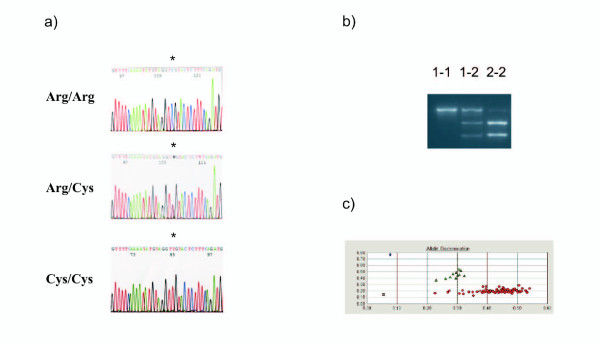
Detection and screening of the TOPBP1*Arg309Cys substitution by (a) direct sequencing, (b) restriction enzyme analysis, and (c) 5'-nuclease allelic discrimination assay. a) Direct sequencing of the sense strand of *TOPBP1 *exon 8. From top to bottom: Homozygous genotype Arg/Arg, heterozygosity Arg/Cys, homozygous genotype Cys/Cys. The position of the mutation is marked by an asterisk. b) Restriction enzyme analysis after incubation with *Tat *I and 2% agarose gel electrophoresis. Lanes from left to right: common homozygote (Arg/Arg, 1-1), heterozygous sample (Arg/Cys, 1-2), rare homozygote (Cys/Cys, 2-2). c) 5'-nuclease allelic discrimination assay identifying common homozygotes (Arg/Arg, red circles), heterozygotes (Arg/Cys, green triangles), and rare homozygotes (Cys/Cys, blue square). NTC, no template control.

**Figure 2:**
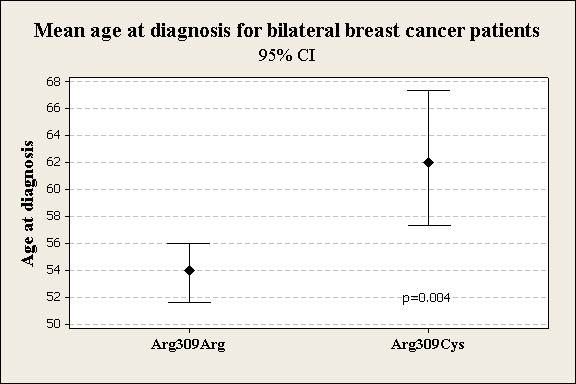
**Mean age at diagnosis of the first primary in patients with bilateral breast cancer stratified by *TOPBP1 *codon 309 genotypes**. The mean is indicated by a black diamond, and 95% confidence intervals are shown.

We confirmed the Arg309Cys missense substitution in an initial sample by restriction enzyme analysis and direct sequencing, and then employed a 5'-nuclease assay for the discrimination of the Arg and Cys alleles in genomic DNA samples from all the HaBCS case-control series (Figure 1). Variant genotype data were obtained for all 1064 patients (1050 with invasive breast cancer, and 14 with in situ carcinoma) and 1014 female population controls. We did not detect significant differences neither in the allelic nor in the genotypic distribution of the Arg309Cys variant between the case and control series under any of the three models applied. Results obtained under a dominant model, with heterozygous and homozygous carriers combined, are presented in Table 1. When stratified by clinical parameters, the most suggestive trend was a slightly lower frequency of the variant in patients with bilateral disease compared with controls (OR = 0.49, 95%CI = 0.24-0.99; p = 0.047) (Table 1), contrary to the expected increase in risk for variant carriers. Furthermore, the mean age at diagnosis of the first cancer among patients with bilateral disease was 62.3 years in carriers and 53.8 years in non-carriers (p = 0.004) (Figure 2). Median age at diagnosis was also higher in the carrier group (p = 0.02). Among patients with bilateral disease, Arg309Cys carriers were underrepresented among patients with nodal metastasis compared with non-carriers (p = 0.01). Carriership also tended to occur at a non-significantly lower frequency in the major group of ductal invasive cancers (OR 0.76, 95% CI 0.56-1.04, p = 0.09). No differences between carriers and non-carriers were observed in the total series with respect to age at diagnosis, hormone receptor or nodal status.

**Table 1:** Arg309Cys genotype distribution in German breast cancer cases and controls

	*TOPBP1 *Genotype			*OR (95% CI) (any Cys vs. Arg/Arg)*	*p*
			
	Arg/Arg	Arg/Cys	Cys/Cys		
**Population controls **(n = 1014)	879 (.87)	130 (.13)	5 (.005)		

**Breast cancer **(n = 1064)	936 (.88)	123 (.12)	5 (.005)	0.89 (0.69-1.15)	0.39

- familial	150 (.88)	19 (.11)	1 (.01)	0.87 (0.53-1.43)	0.56

- bilateral	118 (.93)	9 (.07)	0	0.49 (0.24- 0.99)	0.047

- age < 50 ys	263 (.89)	31 (.10)	2 (.01)	0.82 (0.55-1.22)	0.37

- ductal	606 (.90)	69 (.10)	2 (.003)	0.76 (0.56-1.04)	0.09

- node positive	270 (.88)	36 (.12)	1 (.01)	0.89 (0.61-1.32)	0.63

- ER negative	72 (.87)	10 (.12)	1 (.01)	0.99 (0.51-1.92)	1

Relative distribution of *TOPBP1 *codon 309 genotypes in cases and controls shown as number of carriers and, in brackets, as relative fraction. Odds ratios are calculated for carriers of the rare Cys309 allele (with homozygous and heterozygotes combined) versus common homozygotes. P values were calculated using Fisher's exact test.

Our data indicate that the Arg309Cys substitution occurs at a similar frequency in German individuals as reported in the previous study [8], indicating that its distribution is not confined to the Finnish population. However, we could not confirm the hypothesis that it constitutes a significant risk factor for breast cancer, and the upper limit of our 95%CI is not included within the limits proposed in the Finnish Study (95%CI 1.3-4.2, Ref. [8]). Our study had some 80% power to detect a 1.4-fold difference, thus minor risks cannot be formally excluded. An important difference between the study by Karppinen *et al. *[8] and ours is that the former focussed on familial breast cancer whereas we analysed a hospital-based series unselected for family history. On the other hand, no trend became apparent when the patient subgroup with a first-degree family history of breast cancer in our series was analysed separately. An additional indicator for a genetic predisposition is bilateral disease [9]. However, our results showed that the Arg309Cys variant was rather underrepresented in cases with bilateral breast cancer compared to healthy controls, and we furthermore observed significant differences between carriers and non-carriers with respect to the age at diagnosis which may suggest a protective role for the Cys allele.

Altogether, we find no general association of the *TOPBP1 *Arg309Cys variant with breast cancer risk, and the direction of the marginally significant association with bilateral disease in our study is in conflict with the original data. Our results thus do not support the increased risk for Arg309Cys, though it cannot be ruled out that *TOPBP1 *variants exist which may confer an increased susceptibility towards breast cancer. In the initial report, however, the Arg309Cys substitution was among five different coding variants the only one that appeared associated with breast cancer. From our results, there is no evidence to conclude that the Arg309Cys substitution is a strong cancer susceptibility allele. Further research may reveal whether any *TOPBP1 *gene variants can contribute to hereditary breast cancer risk.

## Abbreviations

**SNP**: single nucleotide polymorphism; **BRCT**: breast cancer 1 carboxyterminal; **PCR**: polymerase chain reaction; **MGB**: minor groove binding.

## Competing interests

The authors declare that they have no competing interests.

## Authors' contributions

MAB carried out the molecular genetic studies, performed the statistical analysis and helped to draft the manuscript. NVB helped to carry out the molecular genetic studies and participated in the design of the study. MB, JHK, and PH participated in its design and coordination and helped in the acquisition of clinical data. TD initiated the study, participated in the analysis and interpretation of data, and drafted the manuscript. All authors read and approved the final manuscript.
